# 3D Characterization of Sponge Cake as Affected by Freezing Conditions Using Synchrotron X-ray Microtomography at Negative Temperature

**DOI:** 10.3390/foods10122915

**Published:** 2021-11-24

**Authors:** Amira Zennoune, Pierre Latil, Fatou-Toutie Ndoye, Frederic Flin, Jonathan Perrin, Christian Geindreau, Hayat Benkhelifa

**Affiliations:** 1 Université Paris-Saclay, INRAE, UR FRISE, 92761 Antony, France; amira.zennoune@inrae.fr (A.Z.); fatou-toutie.ndoye@inrae.fr (F.-T.N.); 2 Université Paris-Saclay, INRAE, AgroParisTech, 75005 Paris, France; 3 Université Grenoble Alpes, Université de Toulouse, Météo-France, CNRS, CNRM, Centre d’Etudes de la Neige, 38000 Grenoble, France; pierre.latil@meteo.fr (P.L.); frederic.flin@meteo.fr (F.F.); 4 Synchrotron SOLEIL, L’Ormes des Merisiers, BP48, Saint Aubin, 91192 Gif sur Yvette, France; jonathan.perrin@synchrotron-soleil.fr; 5 Université Grenoble Alpes, Grenoble INP, 3SR, CNRS, 38041 Grenoble, France; christian.geindreau@3sr-grenoble.fr

**Keywords:** freezing rate, sponge cake, microstructure, synchrotron X-ray microtomography, Cryo-SEM

## Abstract

In this study, the microstructural evolution of a non-reactive porous model food (sponge cake) during freezing was investigated. Sponge cake samples were frozen at two different rates: slow freezing (0.3 °C min^−1^) and fast freezing (17.2 °C min^−1^). Synchrotron X-ray microtomography (µ-CT) and cryo-scanning electron microscopy (Cryo-SEM) were used to visualize and analyze the microstructure features. The samples were scanned before and after freezing using a specific thermostated cell (CellStat) combined with the synchrotron beamline. Cryo-SEM and 3D µ-CT image visualization allowed a qualitative analysis of the ice formation and location in the porous structure. An image analysis method based on grey level was used to segment the three phases of the frozen samples: air, ice and starch. Volume fractions of each phase, ice local thickness and shape characterization were determined and discussed according to the freezing rates.

## 1. Introduction

Sponge cake is one of the most consumed cakes in France. This cake is used as a base for the preparation and the assembly of a large variety of entremets/desserts. The shelf life of this cake becomes a necessity for the bakery industry to face the increasing demand on the retail market. According to a survey on the freezing of pastry products carried out among pastry craftsmen in 2016, sponge cake is the most widely used frozen cake in France. The preparation of sponge cake is divided into two steps: baking and freezing, then thawing and decorating. However, the quality of the sponge cake after thawing can be damaged. Frozen products are likely to undergo physico-chemical changes that could alter their quality. These changes are caused by the evolution of the microstructure (pores and ice crystals size and morphology) during the freezing process due to the formation and growth of ice crystals (crystallization). These modifications influence to a large extent the physical, textural and organoleptic properties of the food resulting in quality deterioration [1,2].

During food freezing, heat and mass transfer occurs between the cold surface and the core, due to temperature and water vapor pressure gradients. It is well known that the freezing rate substantially influences the formation of the microstructure and the subsequent product quality. Thus, fast freezing favors numerous and small ice crystals that lead to low quality changes while slow freezing leads to bigger and fewer crystals that are likely to result in greater deterioration of the quality of frozen products [3]. Beyond food products, freezing rate is also related to quality attributes of freeze-dried pharmaceutical products, particularly to protein denaturation [4,5]. Freezing rate was also found to be critical in the case of cryopreservation of live tissues [6]. Indeed, freezing may damage lipids and proteins and impair cell function and structure.

In the case of porous foods, microstructure includes the presence of pores whose size, number, connectivity and surface play an important role on the heat and water transfer and thus on ice crystallization phenomena. Indeed, vapor diffusion is also present in the pores. The supersaturated vapor is condensed into ice when the freezing point is reached leading to the evapo-condensation phenomena. Understanding these physical phenomena at this scale is not obvious. The few existing works have mainly focused on the effect of freezing rate on quality attributes such as rheology, textural specific volume, baking, staling properties [7,8] or thermophysical properties [9,10,11]. These works tried to relate these properties to the microstructure developed during the freezing process without characterizing this microstructure and the mechanisms involved at the ice crystal and pore scale. In addition, these works are dedicated to bread dough or part-baked bread, and no studies about cake or sponge cake were found in the literature.

In the specific case of sponge cake, the freezing is performed after baking. The classical recipe for a sponge cake consists of 25% wheat flour, 25% sugar, 45% eggs and 5% water [12]. During baking, several transformations and chemical reactions occur, including the Maillard reaction, caramelization of sugar, starch gelatinization, evaporation, crumb formation and coagulation of egg white. The water content is nearly 40% before baking and about 30% after baking. A major part of this water is contained in the starch gel and is chemically bounded to the sugar. The amount of free water available for freezing is small. Studying the formation of ice crystals and pores in such a complex structure is not easy. A non-reactive model sponge cake has been developed by Bousquières et al. [13] where reactive ingredients (flour proteins, sucrose and eggs) were replaced by cellulose derivatives. Several ratios of hydroxypropylmethylcellulose (HPMC) and methylcellulose (MC) were added to native corn starch and water so as to obtain similar apparent viscosity and density of the batter before baking and homogeneity of the crumb cellular structure after baking. Water content of the model sponge cake is higher (70% versus 40% for the real sponge cake); however, the freezing of such a model sponge cake has yet to be studied.

Another limitation for the study of the frozen microstructure is the use of reliable tools. Understanding the microstructure of frozen sponge cake requires a high-resolution technology that allows the visualization of micropores and small ice crystals at negative temperature. Various techniques were used to study crystallization and microstructure evolution during freezing process like microscopy and calorimetry [14,15]. Cryo-scanning electron microscopy (Cryo-SEM) is an imaging method with high resolution that has been used to study frozen food, such as meat [16,17]. This technique requires an important preparation of the samples and therefore may induce structural modifications. Furthermore, Cryo-SEM is a 2D technique that provides incomplete information about the 3D microstructure of the studied sample. Differential scanning calorimetry was used to characterize the thermophysical properties (freezing point, thermal conductivity) of frozen porous food products. However, this method does not provide information about the evolution of the microstructure. X-ray micro-computed tomography (X-ray µ-CT) has been proven to be a reliable method to characterize the 3D microstructure of frozen products [15,18,19,20]. This non-invasive technology makes it possible to follow the dynamics of ice crystal formation and evolution during and after freezing. X-ray µ-CT has been used to study the crystallization of freeze-dried food samples at ambient temperature [21,22,23,24]. However, the study of the microstructure evolution of frozen food is possible in a reliable way only if the sample was preserved from any modification [24]. Several techniques were developed to this end in order to directly analyze frozen food at a negative temperature. Calonne et al. [25] used a thermostated cell (CellStat) mounted on the rotating stage of an X-ray microtomograph to investigate the microstructure of snow samples. Ice cream 3D visualization was performed using X-ray microtomography coupled with a two-stage solid-state Peltier cooling system in order to study crystallization in ice cream at negative temperature [26]. More recently, Vicent et al. [27] developed a new method based on the attenuation coefficient of each constituent of the product and characterized the ice crystals and the 3D microstructure of frozen apple using a cooling stage. Recently, this technique was successfully applied [16,28] to analyze meat [10] and sorbets [22] directly after freezing. These authors used a thermostated box to keep the sample at frozen state during imaging but maintaining the frozen state of the sample during acquisition was limited by time (20 min. maximum). The voxel size was also limited and almost reached 9 µm.

Imaging complex materials with several phases at a high resolution and sufficient contrast may require the use of a synchrotron beamline for microtomography [29]. In the case of conventional laboratory tomographs that use conical X-ray beams, sponge cake samples must be small enough to be close to the X-ray source but large enough so that they do not crumb. To overcome these space constraints, synchrotron X-ray tomography may be employed. Beamlines such as ANATOMIX (Synchrotron SOLEIL, Gif-sur-Yvette, France) offer a wide range of voxel size (from 20 nm to 20 μm), photon energies between 5 and 50 keV that are particularly adaptable for low density materials, such as foods, and a versatile environment to install the experiment’ peripherals [30].

The purpose of this paper is to study the effect of the freezing rate on the microstructure of a model sponge cake using Cryo-SEM and X-ray μCT at high spatial resolution. The samples were scanned at frozen state using the thermostated cell CellStat [25] combined with the ANATOMIX synchrotron beamline. Cryo-SEM and µ-CT image visualization allowed for a qualitative analysis of the ice formation and location in the porous structure. An image analysis method based on grey level was used to segment the three phases of the frozen samples: air, ice and cake matrix. Volume fractions of each phase, ice local thickness and shape characterization were determined and discussed according to the freezing rates.

## 2. Materials and Methods

### 2.1. Samples

#### 2.1.1. Sponge Cake Preparation and Sampling

The model sponge cake was prepared using the following formulation, which contains water, starch and stabilizers [13]:

- Native corn starch (Cargill, Minneapolis, MN, USA), stored in glass bottles at 5 °C;
- Methylcellulose (MC) (Dow Chemical, Midland, MI, USA), type SGA7C, stored in a plastic box at room temperature;
- Hydroxypropylmethylcellulose (HPMC) (Dow Chemical, Midland, MI, USA), type K250M, stored in a plastic box at room temperature;
- Ultrapure water.

The quantities of each ingredient used for the preparation of the sponge cake are summarized in Table 1. The densities are shown for water and native corn starch [31]. By neglecting the effect of stabilizers on the batter density, the theoretical batter density was calculated from water and starch data (Table 1) and was found to be equal to 899 (kg/m^3^).

Model sponge cakes are prepared in two steps: (i) preparation of the cellulose derivatives solution and (ii) preparation and baking of the dough.

Both stabilizers were first mixed and then ultrapure water previously heated to 80 °C was added; the hot water allows a better dispersion of the stabilizers. The solution was then left under strong agitation for 90 min at room temperature. Finally, the solution was placed in a refrigerator at 5 °C for 12 h, under strong agitation in order to allow a complete hydration of the stabilizers to take place.

For the preparation of the dough, the cellulose derivatives solution and the starch were first brought to room temperature. Then the cellulose derivatives solution was whipped for 10 min at speed 10 using a robot (kitchenAid, model 5KSM150, Benton Harbor, MI, USA). The speed was reduced to 2 and the sifted starch was added gradually to the foam for 1 min 15 s to avoid clump formation. After, the mixture was whipped for 2 min to obtain the dough.

For each experiment, 60 g of dough was poured into aluminium molds (10 × 6 × 3 cm^3^) and baked in an electric oven (Whirlpool, Benton, IL, USA) for 30 min at 170 °C. For each preparation, a 100 mL of batter was used to measure batter sponge cake density.

After baking, the sponge cakes were left at room temperature for 45 min (ambient relative humidity RH = 50–60%). They were then individually wrapped in waterproof plastic bags and sealed before being stored in a refrigerator at 5 °C.

#### 2.1.2. Sampling and Freezing

**Figure 1:**
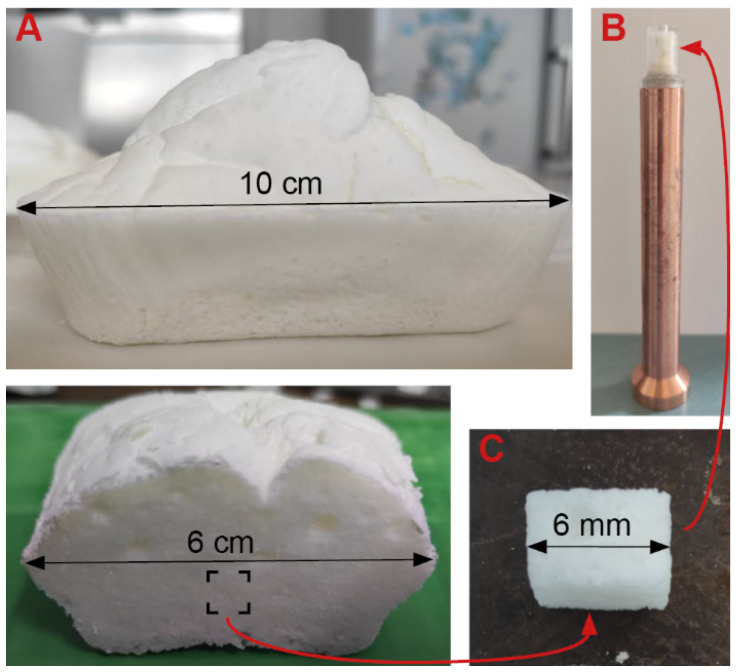
(**A**) Porous model sponge cake (pan dimension 10 × 6 × 3 cm^3^); (**B**) Sponge cake sample on its copper sample holder (enclosed with a PMMA cap); (**C**) Cubic sponge cake sample (6 mm side).

For the experiment, cubic samples (of 6 mm side) (Figure 1C) were cut in the crumb and placed in PMMA-copper sample holders (Figure 1B). The sample and the copper holder were separated by a polystyrene pellet of 5 mm, to improve the homogeneity of the temperature between the top and the bottom of the sample. A droplet of petroleum jelly was used to fix the sample to the polystyrene, which was itself attached to the copper holder using a fixing paste (Pattex, Henkel, Germany). The same paste was used to seal the sample in its PMMA cap, thus insulating the sample from the cell chambers and avoiding water diffusion from the core to the surface.

The sampling was performed in controlled conditions at a temperature of 20 °C and air humidity of 80% in order to avoid moisture loss.

Two freezing rates were applied: (i) fast freezing (17.2 °C min^−^^1^) using an air blast freezer and (ii) slow freezing (0.3 °C min^−^^1^) using a horizontal static freezer. Both freezers were set at −35 °C. For each freezing process, a calibrated type T thermocouple was placed in the center of one reference sample in order to measure temperature evolution during freezing. The thermocouple was attached to a data system (Picolog TC-08, Pico technology, Cambridgeshire, UK) connected to a computer. Four replicates were made for each freezing rate. Samples were directly frozen on the copper sample holder.

### 2.2. Characterization of Thermo-Physical Properties of the Sponge Cake

#### 2.2.1. Density

After batter preparation, the density was determined by weighing (AE 163, Mettler-Toledo, Columbus, OH, USA, precision 10^−4^ g) 100 mL of dough using a measuring cylinder. Two replicates were performed.

After baking, five replicates (5.5 × 5 × 2 cm^3^) were sampled and weighed (about 20 g), then the apparent density was calculated. In order to determine the density of the model sponge cake without air (real density), the same sample was compacted using a vacuum sealing machine (Foodsaver, V2860, Newell Brands, Atlanta, GA, USA) to remove the air from the pores, and the new dimensions were measured to calculate the new volume.

#### 2.2.2. Water Content

Five replicates with dimension size of 6 × 5 × 2 cm^3^ were cut and weighed (about 22 g). Then, the water content was determined according to the NF V03-707 standard by weighing (AE 163, Mettler-Toledo, Columbus, OH, USA, precision 10^−^^4^ g) them before and after drying in an oven (Termaks TS4057, Bergen, Norway) set at 105 °C for 90 min. The water content was calculated according to Equation (1):

(1) $$X_{w}=\frac{m_{0}-m_{f}}{m_{0}}\times 100$$

where $X_{w}$ is the water content of the sponge cake sample, $m_{0}$ is the mass (kg) of the wet sample and $m_{f}$ is the mass (kg) of the dried sample.

#### 2.2.3. Differential Scanning Calorimetry Measurements (DSC)

**Figure 2:**
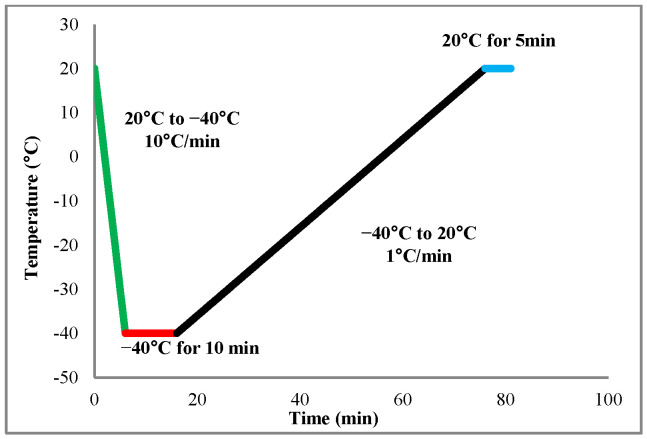
Differential scanning calorimeter protocol.

Differential scanning calorimeter (DSC 1 STARe System, Mettler-Toledo, Columbus, OH, USA) was used to determine the freezing point and the freezable water content. The heat flows and temperature were measured with an accuracy of 0.02 µW and 0.02 °C, respectively. For this experiment, six samples of about 9 mg were cut in controlled conditions of temperature at 20 °C and air humidity of 80% and then placed in an aluminum pan hermetically sealed. For each analysis, the sample was cooled in the pan from 20 °C to −40 °C at 10 °C/min, then held at −40 °C for 10 min, after that, the sample was heated back to 20 °C at 1 °C/min and finally, it was held at 20 °C for 5 min. Figure 2 shows the DSC protocol used for the analysis.

The endothermic ice melting peak obtained, permits to determine the peak temperature ($T_{p}$) and the onset temperature ($T_{o}$), which represents the freezing point. Six replicates were measured.

The freezable water $F_{w}\;\left(\%\right)$ content is calculated using Equation (2) [32]:

(2) $$F_{w}\%=\frac{\Delta H_{w}}{\Delta H_{i}\times T_{w}}\times 100$$

where $\Delta H_{w}$ (J/g) is the total transition enthalpy of ice fusion in the sponge cake sample, $\Delta H_{i}$ (J/g) is the specific latent heat of fusion of ice (334 J/g) and $T_{w}$ (%) is the total water content in the sample.

### 2.3. Cryo-SEM Analysis

For a better interpretation of the µ-CT images, a Cryo-SEM experiment was carried out (Electron Microscopy Facility, IBPS, Paris, France). Both frozen and unfrozen samples were analyzed. In order to avoid thawing during transportation, the samples were frozen on site at −35 °C at a rate of 0.35 °C/min in a small static freezer. Sampling and fracture were directly performed in liquid nitrogen. Fractured surfaces were observed using cryo-SEM (GeminiSEM 500, Zeiss, Oberkochen, Germany) at −120 °C, the pressure in the equipment was 1.6 × 10^−4^ Pa. The accelerating voltage was 3.00 kV or 0.790 kV and the accessible magnification range was from ×13 to ×500,000. Frozen samples were first observed at −120 °C then after partial sublimation at −90 °C for 15 min and 1h.

### 2.4. Synchrotron X-ray Micro-Tomography

#### 2.4.1. Thermostated Cell

**Figure 3:**
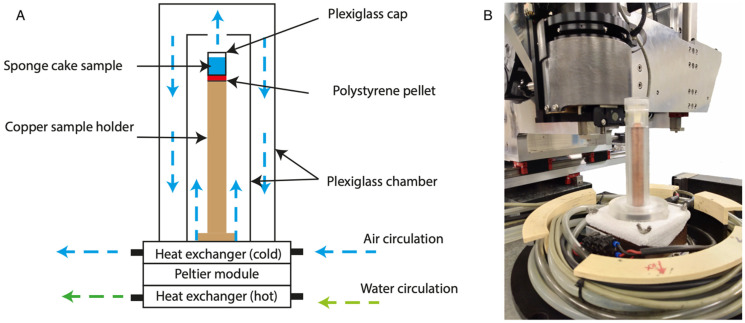
Illustration of the thermostated cell used during the tomographic acquisition: (**A**) CellStat scheme and (**B**) CellStat installed on the ANATOMIX Beamline (Synchrotron SOLEIL, Gif-sur-Yvette, France).

CellStat [25] is a cold cell composed of a Peltier module, which maintains a regulated temperature at the bottom of the copper sample holder described in Section 2.1.2 (Figure 3A). A continuous dry and cold air circulation between the copper sample holder and the plexiglass chambers of the cell homogenizes the temperatures in the chambers and prevents the formation of frost (from the condensation of the room water vapor). In addition, water circulation in the lower heat exchanger allows the dissipation of heat generated by the Peltier module. The system can rotate 360° for single half acquisition modes (Figure 3B).

#### 2.4.2. 3D Image Acquisition

The X-ray microtomography collections were carried out at the beamline ANATOMIX of the Synchrotron SOLEIL (France). The system was operated with a filtered polychromatic (“white”) beam at a central photon energy of 30 keV, optimized with respect to the sample to detector distance (30 mm) and the voxel size of 0.65 µm. 2000 projections were taken over 180° for a total scan time of about 5 min.

CellStat was installed in the ANATOMIX hutch and cooled down to −36°C. Then the frozen samples, already mounted on their copper holder and stored in a domestic freezer (maintained at −30°C), were placed on the thermostated cell (Figure 3B). After 5 min of thermalization, the first scan was launched. Finally, 4 scans were acquired for each sample at different heights (with an overlap between the scans). The total height of the scanned volume is about 5 mm while its diameter is about 1.3 mm.

#### 2.4.3. Image Processing

The µ-CT acquisitions were reconstructed in 3D volumes using PyHST2 software [33] and using the phase contrast Paganin filter [33]. The resulting volume of analysis was: 2048 × 2048 × 2048 vx (corresponding to 1.3 mm side).

**Figure 4:**
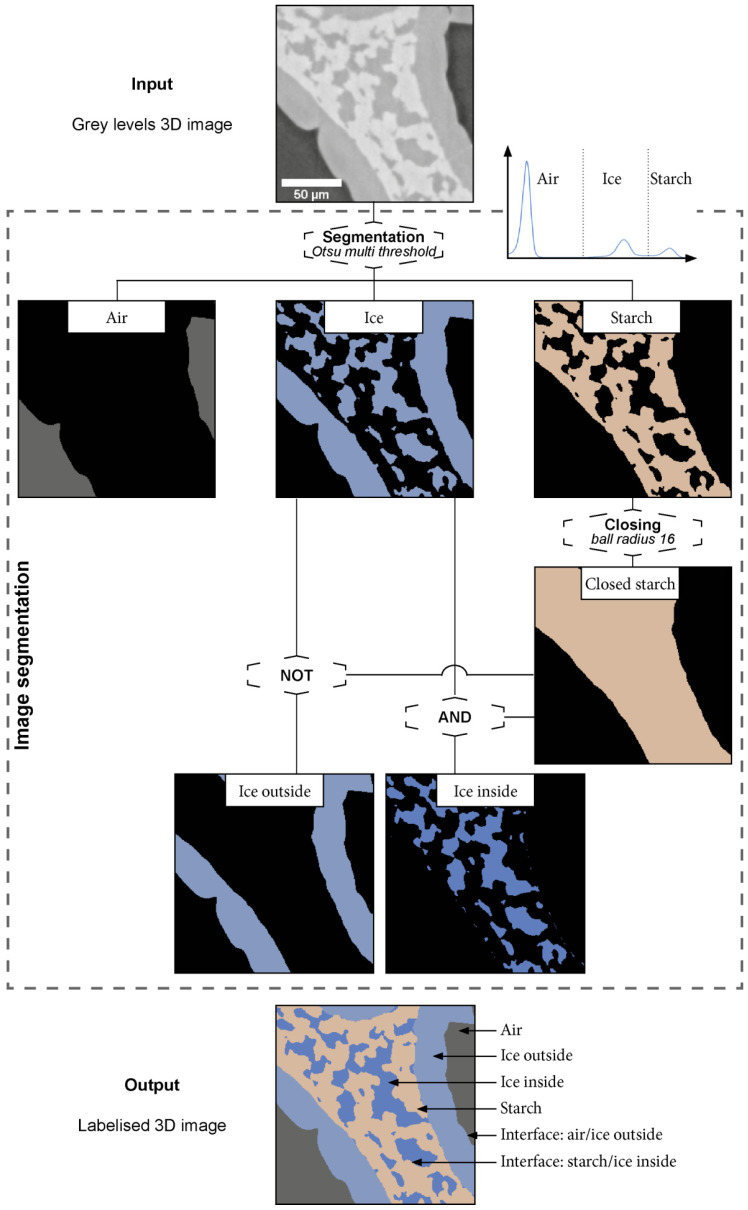
Image segmentation procedure.

The three constituents of the frozen model sponge cake: air, ice and starch are represented by well separated grey levels in the 3D images as shown by the histogram in Figure 4.

The segmentation of these three phases was then simply realized by grey level threshold. By performing a 3D closing operation on the starch phase, it was then possible to create the closed starch phase. This intermediate phase is a “filled” version of the starch phase (Figure 4). The structuring element connects the objects that are closer than its radius into one continuous object. In this work, the optimal radius was 16 voxels. In the following sections, the term matrix will be used to denote the completely closed starch phase with embedded ice crystals.

Finally, the ice phase was segmented into two different phases based on ice crystal location by the mean of logical operations (not/and): (i) the ice inside the matrix (starch + ice inside), defined as the intersection of the ice phase and the starch phase, which is mainly in contact with the starch, and (ii) the ice outside the matrix, which is mainly in contact with the air and located at the surface of the macro-pores of the sponge cake (Figure 4).

#### 2.4.4. Microstructural Description

In this study, the microstructure is characterized by:

▪ The volume of each phase, using a simple voxel counting: $V_{air},\;V_{ice}=V_{ice\;inside}+V_{ice\;outside},\;V_{starch}$, the total volume of the sample is $V_{total}=V_{air}+V_{ice}+V_{starch}$. The volume fraction for each phase is then calculated:

The porosity:

(3) $$\varphi_{Air}=\frac{V_{air\;}}{V_{total}}$$

The ice fraction in the solid phase (without the air):

(4) $$\varphi_{Ice\;in\;the\;solid\;phase}=\frac{V_{ice\;}}{V_{ice}+V_{starch}}$$

The volume fraction of ice outside:

(5) $$\varphi_{Ice\;outside}=\frac{V_{ice\;outside\;}}{V_{ice}}$$

▪ The specific surface area (SSA) is defined by the total surface area of an interface between two phases per the total volume of the sample. It was calculated using MorpholibJ [34] on the one hand, for ice and air interfaces, and, on the other hand, for ice and starch interfaces.
▪ The local thickness of ice inside and outside was computed: it represents the diameter of the largest sphere at a given point that can fit inside the object and containing the given point [35]. This parameter is calculated by the Saito-Toriwaki Euclidean distance transformation algorithm [36]. This algorithm has been implemented as a plugin for Fiji.
▪ The mean curvature: each point of a 3D surface is characterized by two principal curvatures *f_min_* and *f_max_*, which correspond to the maximum and minimum value of the curvature at that point, respectively. The mean curvatures *C* (m^−1^) represent a useful descriptor to characterize the surface shapes [37,38].

(6) $$C=\frac{f_{min}+f_{max}}{2}$$

The sign of the mean curvature indicates the shapes of ice structures. Negative, zero and positive curvature correspond to concave, flat and convex shapes, respectively. In practice, the mean curvatures were obtained using the method proposed by [39,40]. See also [25,41] for additional information.

#### 2.4.5. Statistical Analysis

A statistical analysis (ANOVA) was performed to study the significant effect of the freezing rate on sponge cake microstructure evolution during freezing. The Tukey Kramer test for multiple range comparisons (*p* < 0.05) was used to identify the difference between the measured mean values for volume fractions and SSA.

## 3. Results

### 3.1. Thermophysical Properties of the Model Sponge Cake

#### 3.1.1. Model Sponge Cake Reproducibility

Preliminary preparations of the model sponge cake were carried out in order to verify the reproducibility. The selected properties to compare were the density of batter, the density of sponge cake with air (apparent) and without air (real). These properties were systematically measured for each preparation to ensure their reproducibility and the validity of the model sponge cake. Table 2 shows the average values of the properties of the model sponge cake.

Table 2 shows that the batter measured density is significantly lower than the theoretical one (see Section 2.1.1). During batter preparation, the ingredients are whipped. Air is incorporated in the mix. Thus, the batter measured density includes a fraction of air explaining this lower density value for the batter. During baking, the batter undergoes many physico-chemical changes. These transformations are characterized by cellulose gelation, starch gelatinization, water evaporation and volume expansion of the air due to pore formation, leading to a strong expansion of the cake. In Table 2, the apparent density after baking (392 ± 15 kg/m^3^) is thus significantly lower than density before baking (694 ± 2.2 kg/m^3^). Considering the cake real density even after compaction, a fraction of air seems to still be trapped in the cake since this density of 645 kg/m^3^ is lower than both measured (694 kg/m^3^) and theoretical (899 kg/m^3^) batter densities. This fraction of air, probably contained into the closed pores, does not seem to be extracted during compaction. The mean porosity value mentioned in Table 2 was then calculated considering the ratio between the cake apparent density (containing air) and the batter theoretical density (without air).

Concerning water content, the amount of water used in the preparation is about 75% (64% of ultrapure water and 11% for the starch humidity). After baking, about 60% of this water is still present in the cake. The low standard deviations obtained for porosity and water content after baking confirm the reproducibility of the model sponge cake preparation and validate its use as a model porous food.

#### 3.1.2. Freezing Point and Freezable Water Content

**Figure 5:**
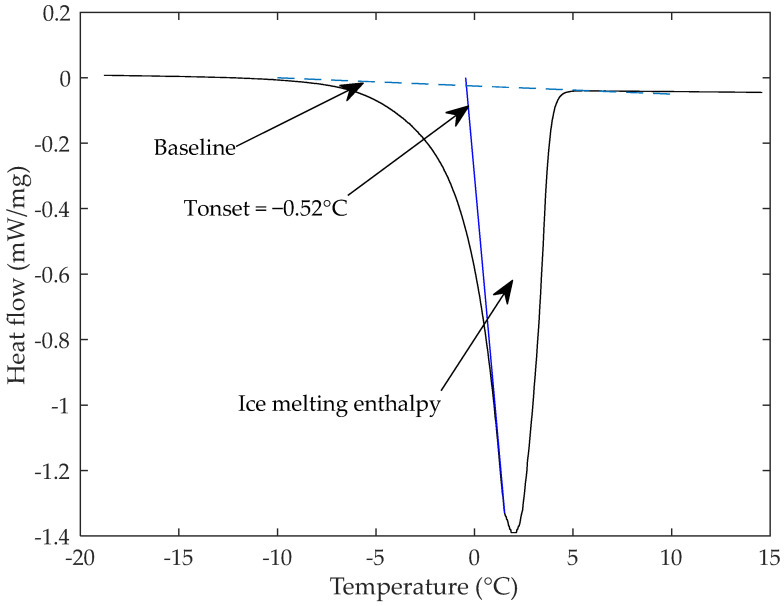
Differential scanning calorimetry curves (DSC) of unfrozen model sponge cake.

The amount of freezable water in model sponge cake was measured using DSC. The samples were directly frozen into the instrument. A typical thermogram of melting is shown in Figure 5. It shows that ice melting started at around −9 °C and that the endothermic ice fusion peak was obtained at around 0.65 °C.

The freezing point represented by the onset temperature was measured by determining the intersection between the tangent and the extrapolated baseline. In the case of this study, the freezing point was −0.52 ± 0.09 °C and the ice fusion peak temperature was 1.98 ± 0.5 °C. The amount of latent heat of fusion absorbed during ice melting was determined by the integral between the heat flow signal and the virtual baseline. The ice melting enthalpy measured was 124 ± 15 J g^−1^. This result was used to calculate the freezable water content using Equation (2). The amount of freezable water was found to be 62 ± 7.5% (*w*/*w*), equivalent to a volume fraction of ice of about 44 ± 5.3%.

### 3.2. Microstructural Image Analysis

Qualitative Analysis

- Ice formation and location

**Figure 6:**
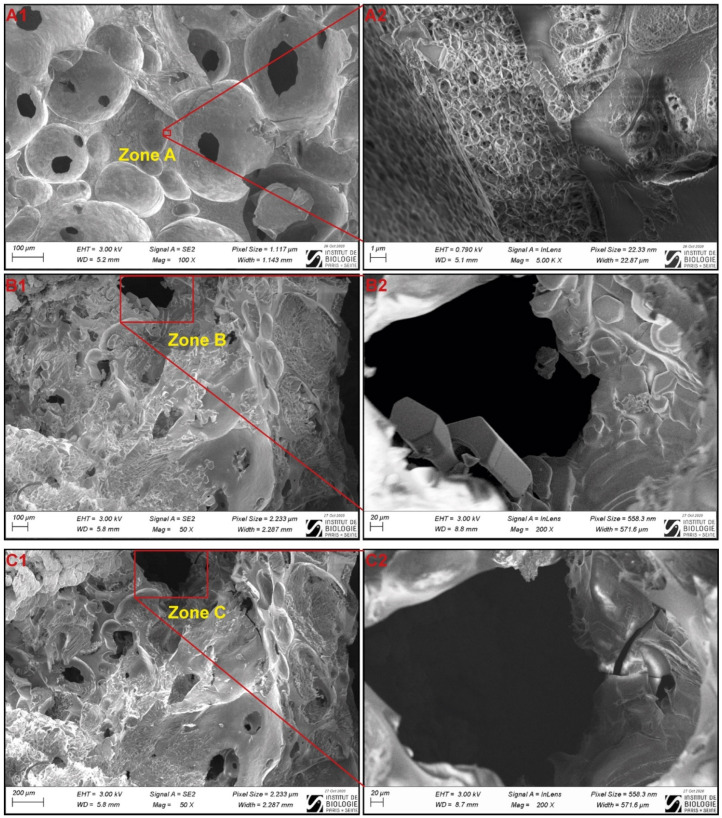
Cryo-SEM images of fractured sponge cake: (**A1**) Unfrozen sample; (**A2**) Zoom on the zone A; (**B1**) Samples frozen for 24 h in a domestic freezer at −35 °C; (**B2**) Zoom on zone B; (**C1**,**C2**) Frozen samples after 1h of sublimation at −90 °C of the images (**B1**,**B2**).

Cryo-SEM was used to visualize the microstructure of model sponge cake before and after freezing (Figure 6). Figure 6(A1,A2) show micrographs of unfrozen samples at magnification of ×100 and ×5000, respectively. They reveal a highly porous microstructure. In these images, the presence of micropores (Figure 6(A1)) and nanopores (Figure 6(A2)) can be noticed in macropores walls. These images also show that the air phase is highly connected. No crystal form is visible and the surface of the pores is smooth and regular.

Figure 6(B1,B2,C1,C2) represent frozen samples of model sponge cake before and after 1 h of sublimation at −90 °C. Figure 6(B1,B2) show the formation of ice crystals all over the pore walls, partially embedded into the starch phase and protrude into the pores. The ice crystals’ length varies between 20 and 100 µm. Sublimation is used to ensure that these specific objects present inside the starch phase and at the pore walls can be reliably identified as ice crystals (Figure 6(C1,C2)). The presence of ice inside the starch phase and at the pores’ walls is therefore confirmed using this Cryo-SEM imaging.

- Effect of freezing rate

**Figure 7:**
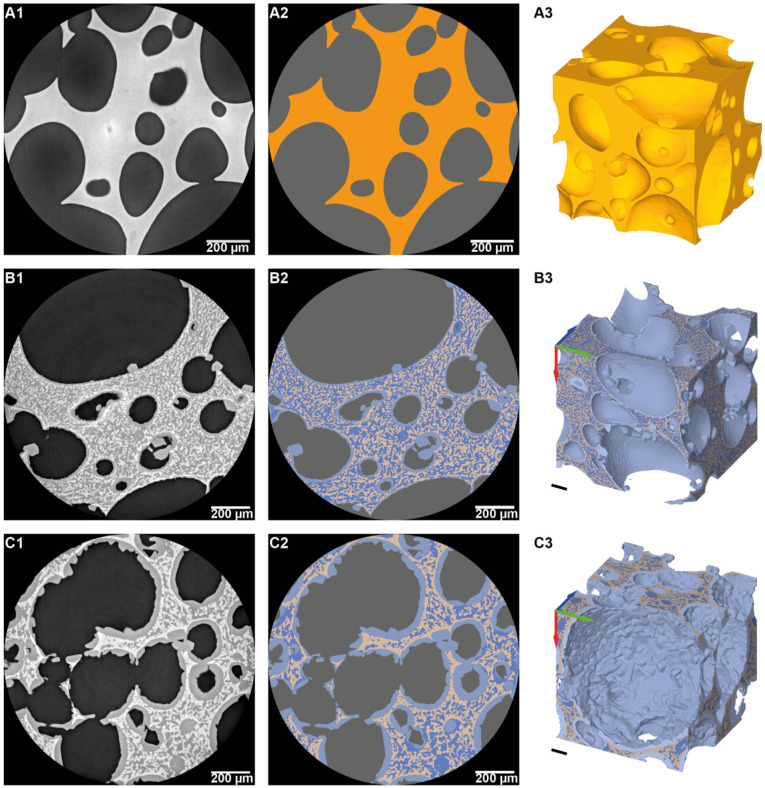
Synchrotron X-ray horizontal slices (grey level images in the left and segmented images in the right) and 3D visualization of the sample after segmentation: (**A1**–**A3**) Unfrozen sponge cake; (**B1**–**B3**) Model sponge cake sample frozen at −17.2 °C min^−1^ (fast freezing); (**C1**–**C3**) Model sponge cake sample frozen at −0.3 °C min^−1^ (slow freezing). (Air in grey, ice outside the matrix in light blue, ice inside the matrix in dark blue and starch in brown). Each cube is 1 mm a side.

Figure 7 presents synchrotron X-ray microtomography images of horizontal slices and 3D representations obtained for unfrozen sample (Figure 7(A1–A3)), for fast frozen samples (Figure 7(B1–B3)) and for slow frozen samples (Figure 7(C1–C3)) before (Figure 7(A1,B1,C1)) and after phase segmentation (Figure 7(A2–C3)).

In Figure 7(A1–A3) no crystalline form is observed and only two materials can be distinguished: the air voids in dark grey and a continuous solid phase (with brightness grey levels) probably consisting of starch, water and hydrocolloids blend. On the other hand, 2D images of the model sponge cake after freezing displayed in Figure 7(B1,C1) clearly shows the presence of a third phase with intermediate grey levels and objects with prismatic/crystalline forms (with sizes and shapes comparable to those obtained during the cryo-SEM observations) that were identified as ice crystals.

Based on the segmentation procedure described on Figure 4, four different phases can be distinguished on the segmented images (Figure 7(A2,B2,C2)): air voids (pores in grey), ice outside the matrix (in light blue), ice inside the matrix (in dark blue) and the starch phase (in brown).

The comparison between Figure 7B,C provides an overview of the effect of the freezing rate on the ice formation (location and size). In the case of fast freezing (Figure 7B), ice formation occurs mainly in the matrix. Ice seems to be evenly distributed, with a fine and homogeneous size. Some cuboid shaped ice crystals and a thin layer of ice are formed at the matrix-air interface. On the other hand, slow freezing (Figure 7C) leads to larger ice crystals with a less homogeneous distribution inside the matrix phase than that observed with fast freezing. Moreover, Figure 7C shows the formation of a thick layer of ice at the interface between the air and matrix unlike the very thin layer obtained for fast freezing.

### 3.3. Quantitative Data Analysis

#### 3.3.1. Representative Sub-Volume Analysis

The high porosity (about 60%) and the presence of large pores (about 1 mm in Figure 7(B1) for example) question the size of the volume that needs to be analyzed for a reliable estimation of the air content.

**Figure 8:**
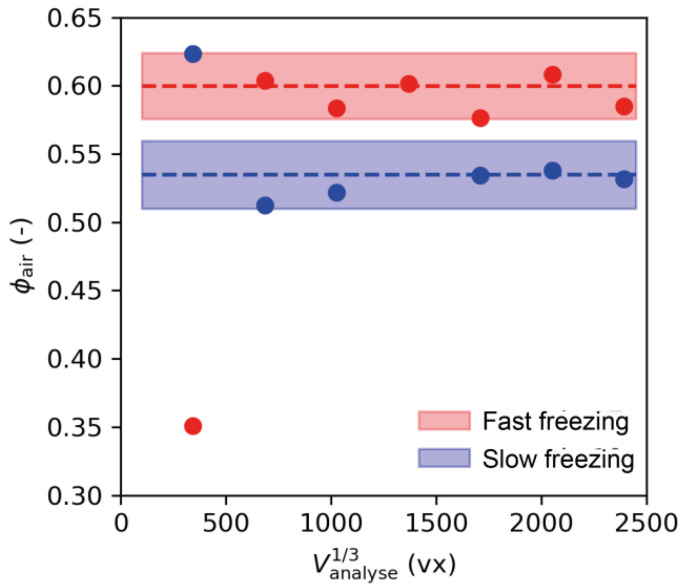
Air volume fractions for different sub-volume samples analyzed to determine the representative sub-volume (colored areas represent standard deviations).

For this reason, mean air volume fraction was calculated for seven different sub-volumes with sizes from 400 × 400 × 400 vx to 2400 × 2400 × 2400 vx (Figure 8), for both a fast and a slow freezing sample (filled dots in Figure 8). The dashed lines represent the average value of the four cropped scans (1500 × 1500 × 1500 vx avoiding boundary effects) and the shaded area is +/− the standard deviation over these four scans.

The mean air volume fractions obtained from the µ-CT image analysis of sub-volumes between 700 × 700 × 700 vx and 2400 × 2400 × 2400 vx were around 60% and 54% for fast freezing and slow freezing, respectively, as shown in Figure 8. These values are in line with the porosity value determined from apparent and real densities measurements (Table 1).

Furthermore, Figure 8 shows no statistical difference between the mean air volume fractions calculated from the sub-volumes higher than 700 × 700 × 700 vx. This result indicates that a sub-volume varying from 700 × 700 × 700 vx to 2400 × 2400 × 2400 vx can reliably be considered as a representative elementary volume for further quantitative analysis. In the following sections, the volumes of the different phases, *V_air_*, *V_starch_*, *V_ice_* and *V_ice inside_*, were calculated using the average of 16 volumes with sizes 1500 × 1500 × 1500 vx. This latter was selected as the representative volume to provide quantitative data of the microstructure of frozen model sponge cake.

#### 3.3.2. Volume Fractions

**Figure 9:**
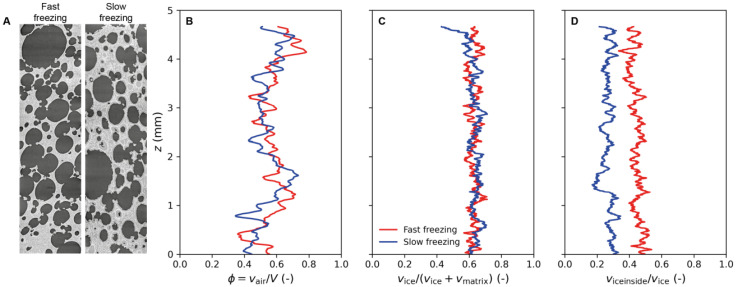
Volume fraction evolution along the height of the sample: (**A**) Vertical slices of grey level images; (**B**) Porosity profile; (**C**) Ice volume fraction (without air) profile; (**D**) Profile of the volume fraction of ice inside the matrix.

As mentioned above, three different phases (air, ice and starch) were segmented based on the grey level contrast as described in Section 2.4.3 (Figure 4), in order to perform quantitative analyses. Figure 9 shows vertical slices in the grey level of samples after fast and slow freezing, respectively (Figure 9A), the corresponding vertical profiles of the porosity (Figure 9B), the volume fraction of ice (without air) (Figure 9C) and the volume fraction of ice inside the matrix (Figure 9D). Table 3 shows the mean volume fractions measured for the different phases: air, starch and ice for both operating conditions: fast and slow freezing.

- Porosity

The porosity for both unfrozen and frozen model sponge cakes was measured. Figure 9B shows the vertical porosity profiles of frozen model sponge cakes. A relative heterogeneity of air content along height can be observed. The porosity profiles are between 40 and 80%, revealing the multiple scale and the natural variations of the porosity. Considering the mean air fractions in Table 3, the unfrozen sponge cake contains about 63 ± 6% of air. This value is close to the one obtained from the density determination shown in Table 2 (56 ± 2%). One can thus consider that the µ-CT imaging provides reliable quantitative information about the sponge cake porosity. The mean volume fraction of air is 62 ± 6% for the fast freezing cake and 57 ± 6% for the slow freezing cake (Table 3), showing a significant difference based on the statistical study (*p* < 0.05). The slight difference between these fractions can be explained by the local heterogeneity of air content in each sample. Nevertheless, in the case of the slow freezing cake, the presence of a thick layer of ice on the pore walls significantly reduces the equivalent diameter of pores and hence the air volume fraction (Figure 7C). This can explained by the air volume fraction being systematically lower in the case of slow freezing rate.

- Ice volume fractions

Considering the profile of the ice volume fraction (without air) shown in Figure 9C, it can be noticed that the ice content is homogeneously distributed along the sample’s height regardless of freezing rate. In addition, the mean total ice volume fractions in Table 3 show that the total ice volume fraction is quite similar for both freezing rates (about 60%). It can be noticed that this value is higher than the ice volume fraction measured using DSC (44%). Indeed, the calorimetry measurements depend on several parameters, particularly in the case of sponge cake in which the water distribution may be heterogeneous. Consequently, the initial amount of water in one sample depends on the sampling location even if, in our case, special care was taken during sampling in order to make sure to sample the different replicates in the same area. In addition, the samples used in DSC are extremely sensitive to the ambient conditions and may undergo dehydration during handling due to their small size (nearly 3 × 3 × 2 mm^3^), even if they were prepared in a controlled (temperature and humidity) atmosphere.

According to Figure 9D and Table 3, the mean volume fraction of ice inside the matrix obtained for fast freezing is significantly (*p* < 0.05) higher (quite twice) than the one found for slow freezing. This ice located inside the matrix corresponds to almost two thirds of the total ice formed in the case of fast freezing. Inversely, for slow freezing, two thirds of the total ice are mainly formed at the air-matrix interface, showing a significant difference (*p* < 0.05) compared to fast freezing. These quantitative results are consistent with the observations described in Figure 7.

#### 3.3.3. Specific Surface Area (SSA) and Local Thickness

Table 4 shows the SSA values of, on the one hand, the interface of ice with the air and, on the other hand, the interface between ice and starch phases for both fast and slow freezing (see Figure 4). It can be noticed that there is no significant difference (*p* < 0.05) between the SSA for the air–ice outside the matrix interface, for both freezing rates. Indeed, an ice layer is always present on macropore walls even if this layer is significantly thicker at a low freezing rate as shown on Figure 7. On the other hand, the SSA value for ice inside the starch matrix interface (see Figure 4) is almost two times more important for fast freezing, where ice is mainly formed inside the matrix, than for slow freezing, as shown in Table 3 and Table 4. The statistical analysis shows a significant difference between fast and slow freezing.

**Figure 10:**
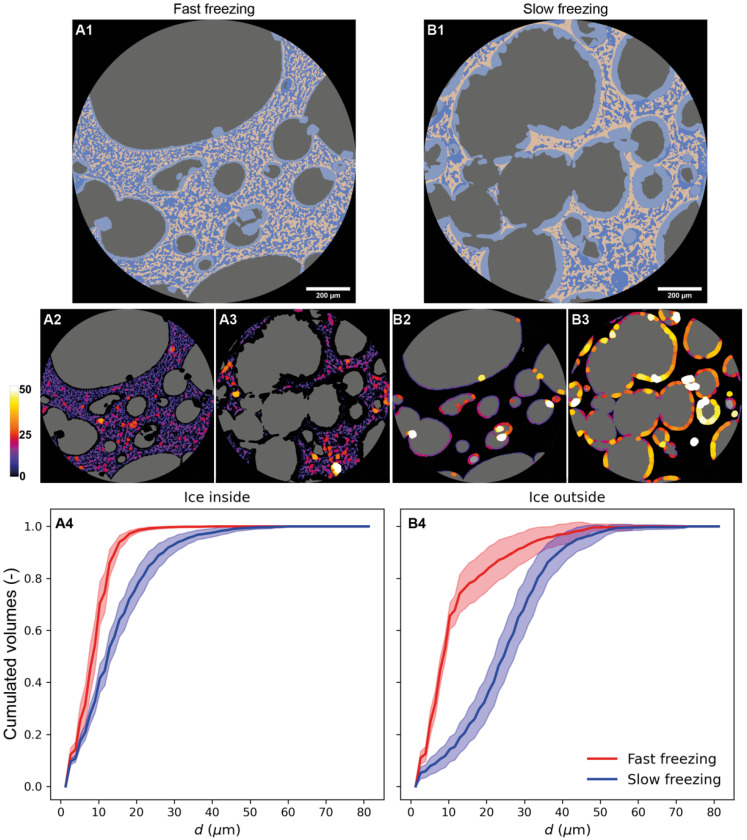
Measurement of local thickness of ice: (**A1**) 2D segmented image of sponge cake sample (fast freezing); (**A2**) Local thickness of ice inside the matrix (fast freezing); (**A3**) Local thickness of ice inside the matrix (slow freezing); (**B1**) 2D segmented image of sponge cake sample (slow freezing); (**B2**) Local thickness of ice outside the matrix (fast freezing); (**B3**) Local thickness of ice outside the matrix (slow freezing); (**A4**,**B4**) Cumulative distribution of local thickness of ice inside the matrix and ice outside the matrix according to the freezing rate.

Figure 10 shows the local thickness (see Section 2.4.4) of ice inside (A2 and A3) and outside the matrix (B2 and B3) for both fast and slow freezing, respectively. The largest ice local thicknesses appear with yellow and white colors while the smallest tend to purple and blue. The cumulative distributions of the ice local thickness diameters are plotted in Figure 10(A4,B4) for ice inside and ice outside the matrix.

Comparing Figure 10(A2) and Figure 10(A3)), it can be seen that the local thickness values of ice inside the matrix were smaller (mainly blue and purple spots in Figure 10(A2)) for fast freezing than the values obtained for slow freezing (more red, yellow and even white spots in Figure 10(A3)). This result was confirmed by the ice distribution displayed in Figure 10(A4). Indeed, the curves of cumulated ice volume show a more homogeneous distribution for fast freezing compared to slow freezing. For fast freezing, 95% of the population of ice inside the matrix is below 20 µm against 45 µm for slow freezing. The larger standard deviations for slow freezing that can be seen in Figure 10(A4) also reflect greater population heterogeneity of ice crystal sizes inside the matrix.

Regarding ice outside the matrix, Figure 10(B2) shows few local thicknesses for fast freezing while in Figure 10(B3), nearly the whole pore walls was lined with colored spots (large thicknesses) for slow freezing. Figure 10(B4) confirms this result. In fact, almost 80% of the local thicknesses of ice outside the matrix were below 10 µm for fast freezing, whereas 80% of the local thicknesses were above 20 µm for slow freezing. The large standard deviations obtained for the large size of fast freezing local thicknesses can be explained by the presence of some individual ice crystals at the pore walls (Figure 10(A1,B2)).

Based on these results, it can be concluded that the slow freezing leads to:

▪ The migration of half the ice content to the pores’ interface with a thick layer of ice (20–30 µm)
▪ A characteristic size of the ice inside the matrix of the same order (20–25 µm)

#### 3.3.4. Mean Curvature

**Figure 11:**
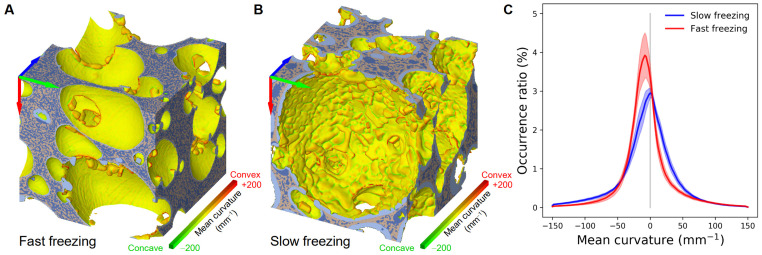
Mean curvature and 3D visualization of sponge cake samples: (**A**) Sponge cake sample frozen at −17.2 °C/min (fast freezing); (**B**) Sponge cake sample frozen at × 0.3 °C/min (slow freezing) (cube of 1 × 1 × 1 mm^3^); (**C**) Mean curvature distributions of a volume of 1 × 1 × 1 mm^3^ regions of interest (ROIs) taken from 16 samples submitted to slow freezing (blue curve). Similarly, the red curve corresponds to ROIs of the same volume taken from 16 fast frozen samples.

Shape characterization was also investigated by calculating the mean curvature in each point of the ice surface for both freezing conditions. 3D visualizations of the frozen model sponge cake samples are shown in Figure 11A,B for fast and slow freezing. The green, yellow and red colors illustrate concave, flat and convex shapes, respectively. The difference between the two freezing rates is obvious in this figure. In the case of the fast freezing, Figure 11A shows a smooth texture, it is globally flat and concave except for the presence of some individual and convex ice crystal shapes. On the contrary, for the slow freezing (Figure 11B), the texture seems to be rough and ice covers the pore walls. Convex, concave and flat surfaces are visible on the walls.

Figure 11C shows the mean curvature distributions of a volume of 1 × 1 × 1 mm^3^ regions of interest (ROIs) taken from 16 samples submitted to slow freezing (blue curve). Similarly, the red curve corresponds to ROIs of the same volume taken from 16 fast frozen samples. The curves are expressed in terms of occurrence ratio, showing the ratio in percentage of the ice surface area exhibiting a mean curvature situated in a particular range of values over the total surface area of the ice agglomerate. The main conclusions from mean curvature comparison (fast and slow freezing) are:

- For fast freezing, the curve is much steeper and high with negative curvature values representing the pore curvatures (e.g., a curvature of −2 mm^−1^ corresponds to a pore diameter of 1 mm). The prismatic ice crystals are characterised by rather flat surface. The graph C in Figure 11 shows that the amount of such surface is of the same order for slow and fast freezing
- For slow freezing, the curve is centred at zero and is much wider. This corresponds to a significant proportion of strong curvatures (concave or convex), which means that the surface is rough with several indentations and bumps.

## 4. Discussion

### 4.1. Microstructure Characterization

Sponge cake samples were visualized using two different imaging techniques (X-ray µ-CT and Cryo-SEM). Qualitatively, synchrotron X-ray µ-CT images provide substantial information about distribution and size of pores (Figure 7). It shows high porosity with a heterogeneous size distribution of macro and microporosity randomly distributed (Figure 7A). The effect of freezing rate can also be described by comparing the fast freezing and slow freezing on the 2D images and 3D visualization (Figure 7B,C). It is noticeable that fast freezing favors the formation of smaller ice particles evenly distributed inside the matrix with the deposition of a thin layer of ice at the air-matrix interface. On the contrary, slow freezing results on the formation of a thick layer of ice in the air-matrix interface and the presence of large ice particles randomly distributed inside the matrix. However, it is worth mentioning that the spatial resolution of 0.65 µm does not allow more detailed information about the presence of nanoporosity and nanoparticles. Indeed, the Cryo-SEM imaging, with a high spatial resolution allowing a magnification of 5000×, clearly shows the presence of nanopores in the wall of macropores of the unfrozen sponge cake (Figure 6(A1,A2)). This nanopore network means that the porosity is highly connected. The Cryo-SEM imaging provides access to useful information about the sponge cake microstructure at this scale. Nevertheless, the Cryo-SEM technique gives only 2D information about the product; therefore, it is not possible to quantitatively analyze the pores and the ice with this technique.

It was made possible by using synchrotron imaging and applying robust segmentation between the different phases. The volume fractions, the ice local thickness and the shape characterization were obtained. But the results must be analyzed considering that the calculated air volume fraction was underestimated since the presence of nanopores could not be detected at a resolution of 0.65 µm and were thus considered as starch.

### 4.2. Effect of Freezing Rate on Ice Formation and Location

Images displayed on Figure 7 and obtained thanks to synchrotron X-ray µ-CT analyses show ice crystal formation after freezing and evidence different microstructures according to the freezing rate. For fast freezing, a fine and homogeneous microstructure was obtained. Conversely, the microstructure resulting from slow freezing was rather coarse and heterogeneous. This result is in line with the well-known effect of freezing rate on solid food products: fast freezing leads to numerous and small ice crystals while slow freezing results in large and fewer ice particles [22,42,43]. Mechanistically, nucleation and growth of ice crystals are controlled by both the thermal resistance and the diffusion of the freezable water into the product. In the case of highly porous food, such as the model sponge cake considered in this work, the mobility and distribution of freezable water is highly constrained by the porosity. In addition to heat transfers, the performance of freezing in such products is principally related to the dynamics of water and ice in both the starch matrix and the pores. This phenomenon is evidenced in Figure 7 where ice at the pore walls can be seen according to the freezing rate. Indeed, a fast freezing rate led to the formation of a thin layer of ice in the pore walls while a thick layer was obtained for slow freezing. In order to reliably assess the effect of freezing rate on ice formation and location, the ice phase was separated into two different parts: ice inside the matrix and ice outside the matrix. This information is, indeed, a key parameter to highlight the difference between fast freezing and slow freezing rate. Ice volume fractions described in Table 3 show that ice was mainly formed inside the matrix for fast freezing (almost 2/3 of the total ice fraction) while most of the ice was located outside the matrix for slow freezing (almost 2/3 of the total ice fraction). These results were confirmed by the SSA and local thickness values presented in Table 4 and Figure 10, respectively. The SSA between ice inside the matrix and the starch is two times less important for the slow freezing rate compared to the fast freezing rate. This result reflects the larger ice crystals formed inside the matrix when the model sponge cake is frozen at a low rate. On the other hand, it is worth noting that SSA values of ice air—outside the matrix interface are similar for both freezing rates, meaning a similar pore wall covering by ice layer. However, the higher local thicknesses described for low freezing rate (Figure 10(B3,B4)) shows that more ice was formed inside the pores when the sample was frozen slowly. Low freezing rate favors the migration of water towards the pores where it crystallizes as evidenced by the volume fractions and the high local thicknesses described in Table 3 and Figure 10(B3,B4), respectively. As mentioned above, the physics of ice formation and distribution during freezing of porous food is highly related to the structural porosity. During freezing, a temperature gradient is applied inside the product. This gradient can lead to moisture migration and water evaporates from the warmer side (matrix) and diffuses through pores due to vapor pressure gradient. This vapor condenses into ice when the freezing point is reached. This phenomenon is called evaporation–condensation. It is also admitted that in the presence of pores, water diffusion is accelerated during freezing inside the product [42]. When fast freezing is applied, the product cools quickly, a high vapor pressure gradient occurs and the local high super-cooling leads to the formation of numerous ice nuclei with a small critical size. Furthermore, the fast freezing rate combined with the low temperature slows down water diffusion resulting in limited growth of nuclei and small ice crystals. Therefore, the diffusive phenomena are limited by the fast freezing rate and the microstructure of the sponge cake does not considerably evolve. On the contrary, during slow freezing, the product cooling is slow and the temperature gradient is low. The nucleation mechanism is limited, due to the high critical size necessary for stable nuclei. This reduces the number of initial ice crystals. At the same time, the low freezing rate favors crystal growth by water diffusion from the liquid phase to the crystal surface and the incorporation of water molecules to the crystal. Considering the thermal diffusivity of the air inside the pores that is much higher (nearly 10^−5^ m^2^/s) than the one of the sponge cake matrix (around 10^−8^ m^2^/s), water have more time to diffuse through the matrix and to evaporate inside the pores. Once the pores’ surface reaches the freezing point, the water vapor inside the pores condenses at the pores’ surface. Ice growth is favored over ice nucleation leading to a thick ice layer at the air–matrix interface.

The formation of ice at the pore walls have been already demonstrated by [43,44], who studied ice formation respectively during bread dough and wheat dough freezing. They noticed that ice formation and growth occurred preferentially in pores sites. Chen et al. [45] studied ice crystal formation and distribution in wheat flour using DSC. They noticed the presence of two endotherms on the DSC curve regardless of freezing rate. The authors admitted that the minor endotherm corresponds to the melting of ice in the small pores within the gluten-starch matrix and the major endotherm to the melting of ice in large pores.

Overall, the freezing rate has a significant impact on the texture of the product. Indeed, the formation of ice inside the pores and in the air–matrix interface can cause the deterioration of the starch granules and of the gluten network in the case of bread [43,44,46,47,48]. These modifications are as important as the ice crystals are large, meaning that they are favored by slow freezing rates. The ruptured gluten network retains gas poorly, hence reducing the loaf volume. The water migration toward the ice crystals and to the crust also promotes the degelatinization of starch and increases the firmness of breadcrumb [49]. Ice formation and growth can damage the pores or merge two adjacent small pores to create a greater one. This affects the microstructure of the sponge cake and hence reduces its quality.

## 5. Conclusions

In this research, a porous model food was analyzed to understand the impact of freezing rate on the microstructure evolution during freezing. Two imaging methods (X-ray µ-CT and Cryo-SEM) were used in this study in order to visualize the microstructure of model sponge cake. A thermostated cell initially developed to study snow was placed on the ANATOMIX synchrotron beamline to analyze the model sponge cake sample at negative temperature and at high resolution. Both methods show the formation of ice at the air-matrix interface and inside the matrix but in different proportions. These results highlight the impact of freezing rate on ice formation and location:

- For fast freezing, 69% of the ice formed during freezing is formed inside the matrix with a homogeneous distribution of small ice crystals;
- For slow freezing, almost 60% of the ice present in the sample is formed at the air-matrix interface; thick ice layers are visible in the pores and the ice is heterogeneously distributed in size and location inside the starch matrix.

The obtained results are of considerable interest and may help to better understand the heat and mass transfer that occur during porous food freezing. Controlling the freezing rate is a key parameter to preserve food quality.

The proposed method and technique can be extended to study the impact of storage conditions on the microstructure evolution. It is important to mention that frozen foods undergo many changes during frozen storage due to storage duration and temperature fluctuations.

## Figures and Tables

**Table 1:** Model sponge cake formulation and ingredient densities.

Ingredients	Quantity (% *w*/*w*)	Density (kg/m^3^)
K250M (HPMC)	0.35	/
SGA7C (MC)	0.46	/
Water	62.64	1000
Starch	36.55	766

**Table 2:** Model sponge cake properties.

Batter Theoretical Density (kg/m^3^)	Batter Measured Density (kg/m^3^)	Cake Real Density (kg/m^3^)	Cake Apparent Density (kg/m^3^)	Porosity (%)	Water Content after Baking (%)
899	694 ± 2.2	645 ± 20	392 ± 15	56 ± 2	60 ± 1.5

**Table 3:** Mean volume fractions of the different phases for unfrozen, fast and slow freezing.

Freezing Rate	Volume Fraction of Air (%)	Volume Fraction without Air (%)			
		Total Ice Fraction	Ice Inside the Matrix	Ice Outside the Matrix	Starch
Unfrozen	63 ± 6 ^a^	/	/	/	/
Fast freezing	62 ± 6 ^a^	62 ± 3 ^c^	43 ± 3 ^d^	19 ± 3 ^f^	38 ± 2 ^h^
Slow freezing	57 ± 6 ^b^	63 ± 1 ^c^	25 ± 5 ^e^	38 ± 5 ^g^	37 ± 1 ^h^

For each column, different letters indicate significant differences between values within a single column, *p* < 0.05.

**Table 4:** Specific surface area SSA (mm^−1^).

Freezing Rate	Interface Air—Ice Outside the Matrix	Interface Ice Inside the Matrix—Starch
Fast freezing	8.4 ± 1.5 ^a^	52.6 ± 12.9 ^b^
Slow freezing	8.0 ± 1.0 ^a^	31.9 ± 11.4 ^c^

For each column, different letters indicate significant differences between values within a single column, *p* < 0.05.

## Data Availability

Not applicable.
